# Does Fractional Anisotropy Predict Motor Imagery Neurofeedback Performance in Healthy Older Adults?

**DOI:** 10.3389/fnhum.2019.00069

**Published:** 2019-02-28

**Authors:** Joost Meekes, Stefan Debener, Catharina Zich, Martin G. Bleichner, Cornelia Kranczioch

**Affiliations:** ^1^Neuropsychology Lab, Department of Psychology, University of Oldenburg, Oldenburg, Germany; ^2^Cluster of Excellence Hearing4All, University of Oldenburg, Oldenburg, Germany; ^3^Research Center Neurosensory Science, University of Oldenburg, Oldenburg, Germany; ^4^Department of Psychiatry, Oxford Centre for Human Brain Activity, Wellcome Center for Integrative Neuroimaging, University of Oxford, Oxford, United Kingdom; ^5^Nuffield Department of Clinical Neurosciences, Wellcome Centre for Integrative Neuroimaging, Oxford Centre for Functional MRI of the Brain, University of Oxford, Oxford, United Kingdom

**Keywords:** motor imagery, EEG, neurofeedback, white matter, fractional anisotropy, MRI, shrinkage linear discriminant analysis

## Abstract

Motor imagery neurofeedback training has been proposed as a potential add-on therapy for motor impairment after stroke, but not everyone benefits from it. Previous work has used white matter integrity to predict motor imagery neurofeedback aptitude in healthy young adults. We set out to test this approach with motor imagery neurofeedback that is closer to that used for stroke rehabilitation and in a sample whose age is closer to that of typical stroke patients. Using shrinkage linear discriminant analysis with fractional anisotropy values in 48 white matter regions as predictors, we predicted whether each participant in a sample of 21 healthy older adults (48–77 years old) was a good or a bad performer with 84.8% accuracy. However, the regions used for prediction in our sample differed from those identified previously, and previously suggested regions did not yield significant prediction in our sample. Including demographic and cognitive variables which may correlate with motor imagery neurofeedback performance and white matter structure as candidate predictors revealed an association with age but also led to loss of statistical significance and somewhat poorer prediction accuracy (69.6%). Our results suggest cast doubt on the feasibility of predicting the benefit of motor imagery neurofeedback from fractional anisotropy. At the very least, such predictions should be based on data collected using the same paradigm and with subjects whose characteristics match those of the target case as closely as possible.

## Introduction

Neurofeedback training based on motor-related brain activity has been proposed as a potential add-on therapy to facilitate post-stroke motor recovery, especially in patients with little or no residual movement (Sharma et al., 2006). In the vast majority of studies to date, the neurofeedback is based on event-related changes in power of the sensorimotor rhythms in the alpha (8–12 Hz) and beta (12–30 Hz) frequency bands of the electroencephalogram (EEG). These changes are termed event-related desynchronization when power decreases and event-related synchronization when power increases (Pfurtscheller and Neuper, 1997; Pfurtscheller and Lopes Da Silva, 1999). A neurofeedback system based on the event-related desynchronization induced by kinesthetic motor imagery provides feedback to the patient regarding the activation of sensorimotor areas without the need of overt movement (Pfurtscheller et al., 1993; Zich et al., 2015a). By this, it can assist the reorganization of neural circuits of the motor system (Chaudhary et al., 2016). Although, stroke may affect motor imagery ability as well as motor execution (de Vries et al., 2011; Feenstra et al., 2016; Oostra et al., 2016), in most stroke patients it is sufficiently conserved to use EEG-based motor imagery neurofeedback (Braun et al., 2017). In fact, there is now a series of studies documenting benefits of motor imagery neurofeedback training in patients with upper limb impairments following stroke (Buch et al., 2008; Broetz et al., 2010; Prasad et al., 2010; Ang et al., 2011, 2015; Caria et al., 2011; Cincotti et al., 2012; Mihara et al., 2013; Ramos-Murguialday et al., 2013; Pichiorri et al., 2015; Zich et al., 2017b—see Cervera et al., 2018 for review).

However, not everyone can successfully use motor imagery neurofeedback. Motor imagery neurofeedback is a type of brain-computer interface. A considerable portion of healthy adults does not achieve the 70% classification accuracy (Guger et al., 2003; Hammer et al., 2012) that is commonly set as the threshold for adequate control of a brain-computer interface (Kübler et al., 2001; Kübler et al., 2004). This inability to control a brain-computer interface has been described as “brain-computer interface illiteracy” (Vidaurre and Blankertz, 2010) or, perhaps more appropriately, “brain-computer interface inefficiency” (Kübler et al., 2011; Hammer et al., 2012). It would be very useful to predict whether a particular individual will be able to use motor imagery neurofeedback, to provide this therapy to only those patients from which we can expect that they can profit from it.

A range of functional, psychological, and neurophysiological measures have been proposed as predictors of motor imagery neurofeedback aptitude (Blankertz et al., 2010; Grosse-Wentrup and Schölkopf, 2012; Hammer et al., 2012; Ahn et al., 2013b; Witte et al., 2013; Bamdadian et al., 2014; Jeunet et al., 2015—see Jeunet et al., 2016 for review), and even more have been shown to be at least associated with motor imagery neurofeedback performance (Nijboer et al., 2008, 2010; Halder et al., 2011; Ahn et al., 2013a; Zich et al., 2015b). However, many of these measures are subject to considerable temporal fluctuations and/or require active engagement from the participant to assess. Halder et al. (2013) proposed the use of anatomical and structural differences to predict motor imagery neurofeedback aptitude^1^. In their study, participants performed motor imagery of two classes of movement which were individually selected out of a total of three classes (left hand, right hand, preferred foot) based on maximum discriminability. A median split on motor imagery neurofeedback performance was used to categorize the participants into high and low aptitude users. While measures based on voxel-based morphometry estimates of white and gray matter volumes had no predictive value, fractional anisotropy of five white matter regions selected using shrinkage linear discriminant analysis correlated significantly with motor imagery neurofeedback performance, and, together with five further regions with weaker correlations, classified high and low aptitude participants with a cross-validation accuracy of 93.75% (Halder et al., 2013). Such accurate prediction of motor imagery neurofeedback aptitude from fractional anisotropy data would be extremely valuable.

However, as with most of the functional measures, the results of Halder et al. (2013) have not yet been replicated. In addition, the motor imagery neurofeedback used by Halder and colleagues differs in two important ways from neurofeedback implementations typically applied in stroke rehabilitation (Cervera et al., 2018). First, the selection of classes for training would normally be based on therapeutic considerations, not on maximum discriminability of the classes. Second, the participants in the study by Halder and colleagues were also considerably younger (mean age 24.5 years) than typical stroke patients. Estimates show that in a country like the United States, almost 75 percent of stroke patients are 65 years or older (Hirtz et al., 2007). Both the neural activation pattern induced by motor imagery (Zich et al., 2015b, 2017b) and the structure and integrity of white matter are known to change with normal aging (Guttmann et al., 1998; Hong et al., 2015; Burzynska et al., 2017). In particular, fractional anisotropy in association fibers declines in older adults (Bender et al., 2016), which might affect their predictive value for motor imagery neurofeedback aptitude. It is therefore currently unclear whether the results obtained by Halder and colleagues generalize to motor imagery neurofeedback in the context of stroke rehabilitation. We aimed to test the approach proposed by Halder and colleagues with motor imagery neurofeedback that is closer to that used for stroke rehabilitation and in a sample whose age is closer to that of typical stroke patients. Our hypothesis was that motor imagery neurofeedback aptitude in older subjects can be predicted from fractional anisotropy using shrinkage linear discriminant analysis.

## Materials and Methods

### Participants

Inclusion criteria for participation were general good health, age ≥ 45 years and native command of German. Exclusion criteria were the presence of any contraindications for magnetic resonance imaging (MRI) as well as current or previous neurological disease. Participants were screened for cognitive impairment using the verbal fluency and trail-making subtests of the German version of the CERAD battery (Consortium to Establish a Registry for Alzheimer’s Disease) (Berres et al., 2000). Twenty-one healthy older adults (mean age 61.4 [48–77] years, 10 females, mean education 11.8 [9–18] years) participated in the study. Three participants reported being left-handed and two described themselves as ambidextrous, though they both used their right hand for writing. This study was carried out in accordance with the recommendations of the Helsinki Declaration of 1975, as revised in 2013. The study protocol was approved by the Ethics Committee of the University of Oldenburg. All participants were informed about the background and procedures of the study as well as about the risks of MRI verbally and in writing. After receiving this information all participants gave written informed consent in accordance with the Declaration of Helsinki.

### Procedure

#### Diffusion-Weighted Imaging

Diffusion-weighted imaging data were acquired on a 3 Tesla Siemens MAGNETOM Verio system (bore diameter 70cm, Siemens AG, Erlangen, Germany). Diffusion-weighted images (20 directions with three repetitions each, b = 1,000 s/mm^2^, 49 slices, voxel size = 1.8 × 1.8 × 2.0 mm^3^, TR = 7,100 ms, TE = 95 ms) were obtained for all subjects.

#### EEG Acquisition

The EEG session took place on a separate day on average 7 days (range 2–17 days) after the MRI session. EEG was acquired from 96 equidistant scalp sintered Ag/AgCl electrodes on an infracerebral electrode cap (EasyCap, Herrsching, Germany) with a nose-tip reference and central frontopolar ground. EEG data were recorded using BrainAmp amplifiers (sampling rate 500 Hz, amplitude resolution 0.1 μV, analog filter 0.015–250 Hz, BrainProducts GmbH, Gilching, Germany).

#### Motor Imagery and Neurofeedback

The motor imagery neurofeedback used in this study has been used previously for at-home motor imagery neurofeedback training in stroke patients (though with mobile amplifiers and fewer electrodes) (Zich et al., 2017a), and in the lab with concurrent functional near-infrared spectroscopy in healthy younger and older participants (Zich et al., 2017b). Here we briefly summarize the processing pipeline, which differs from that of the stroke study only in acquisition hardware and software (mobile vs. lab-based). Presentation of the experimental paradigm, online data recording and processing were performed using OpenVibe (version 0.17.1) (Renard et al., 2010), with the exception of the common spatial pattern analysis which was performed between blocks using EEGLAB (version 12.0.2.4b) (Delorme and Makeig, 2004) running on Matlab, 2012a (Mathworks Inc., Natick, MA, United States).

**FIGURE 1:**
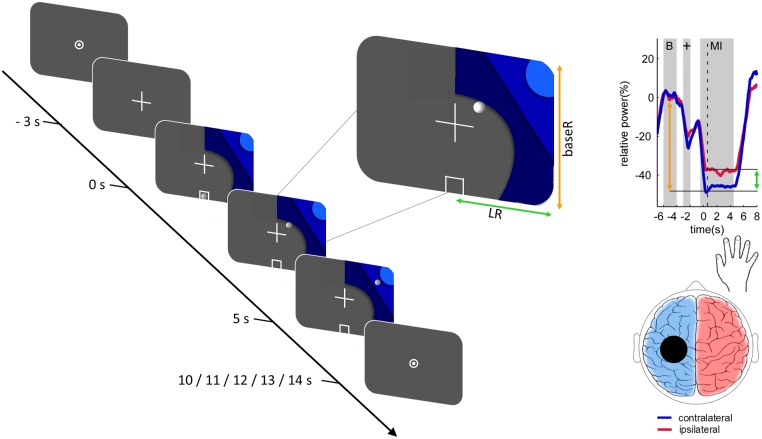
Trial structure and relationship between the 2-dimensional neurofeedback display and motor imagery-induced brain activity. The trial structure is illustrated for a right-hand trial. Each trial was initiated with a fixation-cross and after a delay of 3 s a graphic comprising 3 different shades of blue was added. Onset of the graphic indicated the beginning of the task period (duration 5 s). The location of the graphic indicated which hand to use. During the neurofeedback blocks a white circle resembling a ball moved along the horizontal (green arrow) and vertical (orange arrow) axes according to the classifier output magnitudes. Trials were followed by a fixation dot, resulting in an inter-trial interval of 5–9 s. The relationship between the position of the ball and the motor imagery-induced brain activity at the time point of the dashed vertical line is illustrated on an example time course of the event-related desynchronization. The horizontal ball position is determined by the classification of motor imagery contralateral (blue) vs. ipsilateral (red), as illustrated by the green arrow. The vertical ball position is determined by the classification of contralateral baseline (“B”) vs. contralateral motor imagery, as illustrated by the orange arrow. Reproduced, with permission, from Zich et al. (2017a).

All participants completed two blocks of motor imagery with concurrent EEG recording. Each block consisted of 20 trials for each hand in quasi-random order, for a total of 40 trials per block. Each trial started with a 5 s baseline (“rest”), followed by a 3 s cue signaling the motor imagery was about to start, and a 5 s motor imagery period. Trials were separated by a quasi-random interval of 0–4 s. During the motor imagery period, participants were instructed to perform kinesthetic motor imagery of repeated hand closing and opening with either the right or the left hand, as indicated by a visual cue (see Figure 1). Participants received extensive, standardized instruction regarding the motor imagery task and then practiced offline until the experimenter was satisfied they understood the task.

The first block of motor imagery was completed without neurofeedback. Subject specific neurofeedback parameters were derived from these data. To this end, EEG signals from the central 35 channels were filtered (8 Hz high-pass, 30 Hz low-pass, Hamming-windowed) and epoched to left, respectively, right motor imagery (0.5–4.5 s after trial onset). After joint probability rejection of artifactual epochs (6 standard deviations for individual channels, 2 standard deviations for global activity), the remaining data were submitted to common spatial pattern analysis (Ramoser et al., 2000). By maximizing the variance of the signal for one class (e.g., left hand motor imagery) while simultaneously minimizing the variance of the signal for a second class (e.g., right hand motor imagery), common spatial pattern analysis is a computationally efficient way to obtain spatial filters optimized for detection of power differences between two classes. Since common spatial pattern analysis is based on individual neurophysiological data, it accounts for interindividual differences while also enhancing the signal-to-noise ratio and it is therefore commonly applied in motor imagery neurofeedback paradigms (Blankertz et al., 2008).

Common spatial patterns were calculated for the contrast of left vs. right motor imagery. For each side, the most neurophysiologically plausible filter was selected from among the three filters with the highest variance segregation for that side (i.e., lowest variance) and the filter coefficients of the two selected common spatial patterns were passed on to OpenVibe. In OpenVibe data from the first block were spatially filtered using the selected common spatial patterns and temporally filtered using a 4th-order Butterworth band-pass filter (8–30 Hz, 0.5 dB pass band ripple) and epoched to left-hand motor imagery (0.5–4.5 s), right-hand motor imagery (0.5–4.5 s), and baseline (-7–3 s) epochs. Each epoch was then subdivided into 49 overlapping 1-s bins each shifted by 62.5 ms. Three classifiers were trained: left motor imagery vs. baseline (BaseL), right motor imagery vs. baseline (BaseR), and left motor imagery vs. right motor imagery (LR). To train each classifier, logarithmic average band power for all epochs of the respective classes was submitted to linear discriminant analyses with sevenfold cross-validation and the mean classifier was used to provide neurofeedback in the second motor imagery block. During the second block individuals performed exactly the same task, but now real-time EEG-based neurofeedback was provided by means of a white ball moving on the screen (see Figure 1). To realize this, incoming data were spatially filtered using the selected common spatial patterns, band-pass filtered and the logarithmic band power of 1-s bins (offset of 62.5 ms) classified. The vertical movement of the ball was controlled by the baseL and baseR classifiers for left and right motor imagery trials, respectively. Horizontal movement was controlled by the LR classifier (see Figure 1; Zich et al., 2017b). Participants were encouraged to move the ball to the top left or right corner, depending on the side of the trial.

### Data Processing

#### Preprocessing of Diffusion-Weighted Images

Diffusion-weighted images were analyzed using ExploreDTI (version 4.8.6) (Leemans et al., 2009). Raw images were corrected for scanner drift, subject motion and eddy current distortions. Fractional anisotropy maps were then exported. To avoid interpolation of fractional anisotropy values, the ICBM Mori template (Mori et al., 2008; Oishi et al., 2008) was warped to each of the individual fractional anisotropy maps by estimating normalization parameters from the fractional anisotropy map to MNI space using SPM 12 (Friston et al., 2007), inverting the parameters, and then applying these inverted parameters to the ICBM Mori white matter template. Mean fractional anisotropy for all voxels with an fractional anisotropy > 0.25 was extracted for each of the 48 ICBM Mori atlas regions.

#### Offline EEG Preprocessing

EEG data were offline processed using EEGLAB (version 14.1.1) (Delorme and Makeig, 2004) running on Matlab, 2016a (Mathworks Inc., Natick, MA, United States). High-pass (1 Hz) and low-pass (40 Hz) Hamming-window filters were applied to the continuous EEG data. Channels with a temporal variance > 3 standard deviations above the mean temporal variance, where both mean and standard deviation are calculated across all 96 channels, were flagged as potential bad channels and removed if visual inspection confirmed them as bad channels (mean [range] number of channels removed: 0.57 [0–4]). The continuous data was then split into 1-s non-overlapping epochs (Stropahl et al., 2018). Epochs containing major artifacts were rejected using first thresholding (±500 μV) and then joint probability rejection (6 standard deviations for individual channels, 2 standard deviations for global activity). The remaining epochs were submitted to independent component analysis (Bell and Sejnowski, 1995). Components reflecting eye movements, heart activity and reference artifacts were selected by visual inspection of component maps and component time courses and marked for later rejection. The independent component decomposition and bad channel information were then copied to the original (continuous and unfiltered) dataset. Bad channels (same channels as removed before independent component analysis) were eliminated and components previously marked for rejection were removed. Removed channels were then interpolated. The cleaned EEG data was then entered into the same pipeline for neurofeedback processing described above (section Motor Imagery and Neurofeedback). The only differences between the online and the offline analyses were therefore in the interpolation of bad channels and the removal of artifactual independent components in the offline analysis.

### Statistical Analysis

#### Motor Imagery Neurofeedback Performance

For each participant, three raw performance measures were calculated as follows^2^:

(1) $$BaseL=100*\frac{\text{​}C_{B}+C_{MI\;Left}}{T_{B}+T_{MI\;Left}}$$

(2) $$BaseR=100*\frac{\text{​}C_{B}+C_{MI\;Right}}{T_{B}+T_{MI\;Right}}$$

(3) $$LR=100*\frac{\text{​}C_{MI\;Left}+C_{MI\;Right}}{T_{MI\;Left}+T_{MI\;Right}}$$

where *C_x_* represents the number of correctly classified segments for condition x (e.g., *C_MILeft_* is the number of left motor imagery segments classified as left motor imagery), and *T_x_* represents the total number of segments for condition x. In other words, BaseL performance was defined as the number of baseline segments classified as baseline plus the number of left motor imagery segments classified as left motor imagery, expressed as a percentage of the combined total number of baseline and left motor imagery segments. BaseR performance was similarly defined but substituting right motor imagery for left motor imagery. LR performance was defined as the number of left motor imagery segments classified as left motor imagery plus the number of right motor imagery segments classified as right motor imagery, again expressed as a percentage of the combined total number of left motor imagery and right motor imagery segments.

Since the aim was to obtain an upper estimate of the general ability of each participant to control their motor imagery-related brain signals, further analyses only used the maximum motor imagery neurofeedback performance, i.e., the best performance per participant across the three classifiers. For example, if a participant had BaseL performance = 85%, BaseR performance = 82% and LR performance = 88%, their maximum motor imagery neurofeedback performance was 88%. Note that this is slightly different from the approach by Halder and colleagues, who used a calibration block without feedback to select the two classes with the best discriminability out of three total classes and then determined performance in a test block that consisted only of trials of these two classes. Finally, participants were categorized as high and low performers based on a median split on overall motor imagery neurofeedback performance. This procedure, including the median split, was applied separately for online and offline performance.

#### Performance Prediction

Statistical analyses were performed using R version 3.5.0 (R Core Team, 2018). Shrinkage linear discriminant analysis (SLDA) was performed using the “sda” package (version 1.3.7) (Ahdesmaki et al., 2015). Our primary analysis repeated the procedure used by Halder et al. (2013), using shrinkage linear discriminant analysis to predict online motor imagery neurofeedback performance by including mean fractional anisotropy for each of the 48 white matter regions in the ICBM Mori atlas (Mori et al., 2008; Oishi et al., 2008). Variable selection for shrinkage linear discriminant analysis was based on cross-validating correlation-adjusted *t*-scores (Zuber and Strimmer, 2009) in the training sample. Variables (for our primary analysis these were mean fractional anisotropy values for each of the white matter regions; see also Table 1) with an average correlation-adjusted *t*-score > 4 (corresponding roughly to a *p*-value of 0.1) in cross-validation were included. In a second step, we used the results of this analysis to predict offline performance. For comparison we repeated the shrinkage linear discriminant analysis using only the regions that were reported as contributing to prediction of online performance by Halder et al. (2013).

**Table 1:** Overview of shrinkage linear discriminant analyses.

Analysis	MI-NF performance	White matter regions^a^ included	Other variables included	Variables selected	Prediction accuracy	*p*-value
1	Online	48 regions^b^	–	Column and body of fornixLeft anterior corona radiata	84.8%	0.045
2^c^	Offline	Column and body of fornixLeft anterior corona radiata	–	All	53.9%	0.532
3	Online	5 regions^d^	–	All	29.9%	0.934
4	Online	10 regions^e^	–	All	32.7%	0.915
5	Online	48 regions^b^	Age Education Gender Handedness Age:Education^f^ VF-A VF-S TMT-A TMT-B	Middle cerebellar peduncleLeft anterior corona radiata Age	69.6%	0.496
6^g^	Offline	Middle cerebellar peduncleLeft anterior corona radiata	Age	All	55.6%	0.312
7	Online	5 regions^d^	Age	All	39.4%	0.790
8	Online	10 regions ^e^	Age	All	42.8%	0.687

**FIGURE 2:**
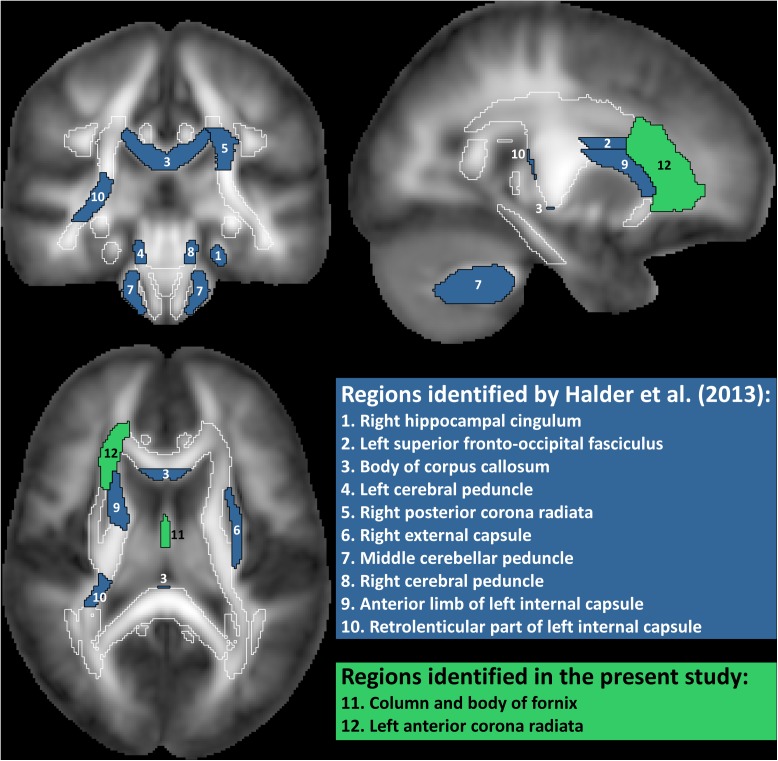
White matter regions identified as predictive of motor imagery neurofeedback performance by Halder et al. (2013) and in the present study. Regions filled in blue are from the study by Halder et al, where regions 1**–**5 showed significant correlations with motor imagery neurofeedback performance and regions 6–10 contributed to prediction in a high proportion of cross-validation folds but the correlation with performance was not statistically significant. Regions filled in green are from the present study. Correlation of FA value in the fornix with performance was only significant if age was not included as additional predictor. Total white matter is outlined in white.

*^a^Defined by the ICBM Mori atlas (Mori et al., 2008; Oishi et al., 2008). ^b^i.e., all the regions in the ICBM Mori atlas. ^c^This analysis uses the regions selected in analysis 1. ^d^The five regions which showed significant correlations in the study by Halder et al. (2013), i.e., regions 1–5 in Figure 2. ^e^The ten regions most frequently selected in the study by Halder et al. (2013), i.e., regions 1–10 in Figure 2. ^f^i.e., the interaction effect of age by education. ^g^This analysis uses the variables selected in analysis 5. MI-NF, Motor imagery neurofeedback; VF-A, Verbal Fluency-Animals; VF-S, Verbal Fluency-S Words; TMT-A, Trail-Making Test part A; TMT-B, Trail-Making Test part B.*

Prediction accuracy was derived from ten repeats of fivefold cross-validation. Statistical significance was assessed by permuting the dependent variable across all participants (10,000 Monte Carlo permutations per analysis), since the commonly used binomial test has been shown to have an inflated rate of type I errors in this type of analysis (Noirhomme et al., 2014). If the proportion of simulations with a cross-validation accuracy greater than or equal to the observed accuracy was smaller than 0.05, the result was considered statistically significant (i.e., α = 0.05).

## Results

### Motor Imagery Neurofeedback Performance

Online performance was best for the BaseL classifier in 12 participants, for the BaseR classifier in 8 participants, and for the LR classifier in 1 participant. Offline performance was best for the BaseL classifier in 6 participants, for the BaseR classifier in 10 participants, and for the LR classifier in 5 participants. Median online overall motor imagery neurofeedback performance was 78.2% (range 50.6–94.0%). Median offline overall motor imagery neurofeedback performance was 67.8% (range 52.8–83.4%). Neither online performance (Pearson *r* = 0.10, *p* = 0.673) nor offline performance (Pearson *r* = 0.01, *p* = 0.979) correlated significantly with age.

### Shrinkage Linear Discriminant Analysis

**FIGURE 3:**
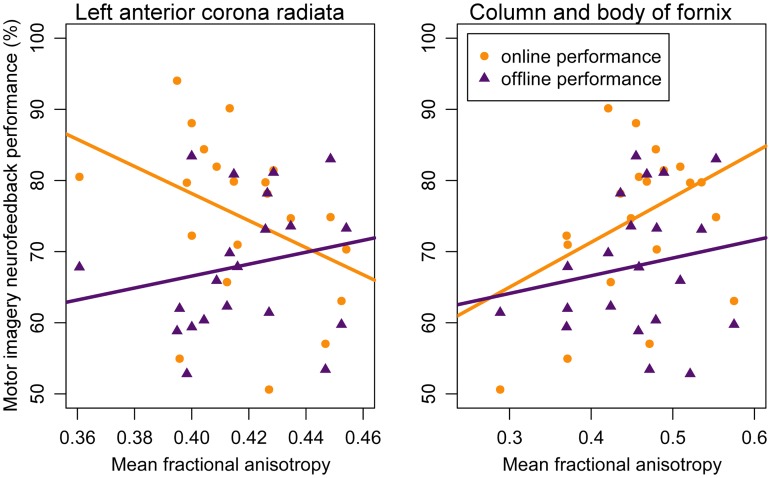
Correlation between motor imagery neurofeedback performance and mean fractional anisotropy in the left anterior corona radiata (left panel) and mean fractional anisotropy in the column and body of the fornix (right panel). Lines represent regression lines with motor imagery neurofeedback performance (yellow: online; purple: offline) as criterion and mean fractional anisotropy value as predictor. Significant partial correlations between mean fractional anisotropy were found for online performance but not for offline performance – see text for details.

*Analysis 1* Our primary analysis, i.e., shrinkage linear discriminant based on all white matter regions (see Table 1, analysis 1), significantly predicted group membership (low vs. high performers) with an accuracy of 84.8% (permutation test: *p* = 0.045, one-sided; note that the corresponding *p*-value was considerably larger than the *p*-value that would be obtained using a binomial test as used by Halder and colleagues: 17/21, *p* = 0.004, one-sided). The regions selected for predicting online accuracy were the column and body of the fornix (partial correlation = 0.47, *p* = 0.037) and the left anterior corona radiata (partial correlation = –0.54, *p* = 0.015) (regions 11 and 12 in Figure 2; see also Figure 3). Neither of these regions was among the ten regions selected for prediction by Halder et al. (2013).

*Analysis 2* Using the two regions selected for the prediction of online performance to predict offline performance (see Table 1, analysis 2) – i.e., low vs. high performers split on the median of offline accuracy—yielded a non-significant prediction accuracy of 53.9% (permutation test: *p* = 0.352). Average fractional anisotropy in neither the column and body of the fornix (partial correlation = 0.15, *p* = 0.541) nor the left anterior corona radiata (partial correlation = 0.16, *p* = 0.505) correlated significantly with offline performance (see Figure 3).

*Analysis 3 and 4* Using the regions that showed significant correlations in the Halder et al. study (regions 1–5 in Figure 2; see Table 1, analysis 3) predicted online group membership with 29.9% accuracy (permutation test: *p* = 0.934). Extending the predictor set to all of the most frequently selected features in the Halder et al. study (regions 1–10 in Figure 2; see Table 1, analysis 4) slightly increased the accuracy of online group membership prediction to 32.7% but also did not reach significance (permutation test: *p* = 0.915).

*Analysis 5–8* Age-related changes in cognitive performance are known to correlate with fractional anisotropy in many white matter tracts—in particular the fornix (Burzynska et al., 2017; Hayek et al., 2018), one of the two regions selected as predicting online group membership in ur primary analysis. In fact, *post hoc* analyses indicated that in our sample, age was significantly correlated with fractional anisotropy in the fornix (Pearson *r* = –0.49, *p* = 0.025) though not with fractional anisotropy in the left anterior corona radiata (Pearson *r* = -0.35, *p* = 0.112), the second selected region. To assess whether such correlations with age in particular, or effects of other demographic variables that are known to be associated with white matter structure (gender, education, handedness) masked associations with the regions identified by Halder and colleagues, we ran all shrinkage linear discriminant analyses again but included these additional (candidate) predictors as well as scores on the CERAD subtests (see Table 1, analysis 5). The resulting model predicted group membership with 69.6% accuracy but this was not significantly better than chance according to permutation testing (*p* = 0.496; note that a binomial test would have reached significance under the most favorable interpretation: 15/21, *p* = 0.039, one-sided). The predictors selected for prediction were mean fractional anisotropy in the middle cerebellar peduncle (partial correlation -0.30, *p* = 0.204) and the left anterior corona radiata (partial correlation -0.36, *p* = 0.124) as well as age (partial correlation 0.13, *p* = 0.591). None of the other variables showed an association with offline performance. Using the three variables selected in this analysis to predict offline performance group membership (see Table 1, analysis 6) decreased accuracy to 55.6% (permutation test: *p* = 0.312). Finally, using the five regions with significant correlations in the Halder et al. study plus age (see Table 1, analysis 7) yielded a prediction accuracy of 39.4% for online group membership (permutation test: *p* = 0.790). Including the additional five regions reported by Halder et al. as frequently selected for prediction (see Table 1, analysis 8) increased prediction accuracy for online group membership only slightly to 42.8% (*p* = 0.687).

## Discussion

Our primary analysis showed that for a group of older healthy participants fractional anisotropy could be used to distinguish good from poor performers in a motor imagery neurofeedback paradigm. This appears to be in line with the idea that fractional anisotropy can predict motor imagery neurofeedback aptitude (Halder et al., 2013). However, the brain structures that were predictive for the our sample differed from those in the aforementioned study. Conversely, the structures identified in that study did not yield predictions for our sample that were significantly better than chance. Moreover, within our sample, prediction for online performance did not generalize to offline performance. Adding age to our primary analysis resulted in loss of significance according to permutation testing, but adding age to the other analyses did not substantially change the results.

What does this mean for the use of fractional anisotropy values to predict motor imagery neurofeedback aptitude? Despite using very similar parameters for EEG and MRI acquisition and closely following their data processing pipeline—albeit using alternative software implementations – we find a completely different set of white matter regions to be predictive of motor imagery neurofeedback performance than did Halder et al. (2013). Our results are based on more subjects and more classifications per subject to calculate performance than those by Halder et al. (2013), and therefore clearly indicate that one cannot simply take the predictors previously identified and apply them to any motor imagery neurofeedback setting, without regarding the details of the paradigm or the characteristics of the participants.

One reason for the lack of convergence between the present results and those reported by Halder and colleagues could be that the precise regions that can be used to predict motor imagery neurofeedback performance vary with the motor imagery task or other aspects of the paradigm. The regions identified by Halder and colleagues may therefore not reflect a general aptitude for motor imagery neurofeedback, but rather specific ability in the tasks used in their study. Different tasks will usually activate different cortical areas. Fractional anisotropy in particular white matter tracts may be related to the activity of the cortical areas they are connecting. Hebbian learning suggests that if two areas show strong concurrent activity the connection between those areas should be strengthened, i.e., through increased myelination which should correspond to higher fractional anisotropy (Fields, 2015). It might also be speculated that cortical areas connected to white matter tracts with high fractional anisotropy are easier to activate voluntarily. Regardless of the underlying mechanism, in this scenario different white matter tracts would be predictive of motor imagery neurofeedback aptitude in the study by Halder et al. (2013) than in the present study because the motor imagery tasks differ between the two studies. If so, the regions identified in the present study, i.e., fornix, left anterior corona radiata, and middle cerebellar peduncle) should be functionally involved in hand motor imagery. While the fornix may reflect a learning component given its connections to the hippocampus, such an association should be task-independent. In any case such an interpretation seems tenuous given the limited number of trials. Damage to the left anterior corona radiata has been associated with poorer motor imagery ability in stroke patients (Oostra et al., 2016) but without evidence for a role in hand motor imagery specifically. The middle cerebellar peduncle, finally, has been implicated in motor imagery (Sacheli et al., 2017) and motor impairment (Liang et al., 2009) but only in the context of gait, rather than hand movement. In other words, based on the available evidence, none of the three regions identified in the present study appears to be specific to hand motor imagery. Task specificity is hence unlikely to be the major explanation for the discrepancies between our results and those of Halder and colleagues. Conversely, we did not find associations with regions that one would have expected. In particular, neither large associative tracts such as the superior fronto-occipital fasciculus, nor tracts connecting to cortical areas known to be involved in motor execution and imagery, such as the posterior limb of the internal capsule and parts of the corpus callosum (Imfeld et al., 2009; Vergani et al., 2014) demonstrated predictive value. One possible explanation for the lack of large associative tracts in the set identified here may be that such tracts serve many functions, and so although the particular fibers relevant for motor imagery might show some correlation, this is relationship is masked by the “noise” of the many other fibers that run in these tracts. The absence of tracts connecting cortical areas involved in motor imagery may in fact be explained by task specificity: if specific tracts are predictive only for a particular task (e.g., right-handed motor imagery) then their influence may be diluted if other tasks (e.g., left-handed motor imagery) are included in the same paradigm.

Rather than young adults as in the vast majority of motor imagery neurofeedback studies, we specifically recruited healthy older adults (mean age 61.4 years) in order to match the age of our sample to the target population for motor imagery neurofeedback rehabilitation. There is some evidence that both the age-related decline in fractional anisotropy and its association with cognitive function are particularly prominent for the fornix (Burzynska et al., 2017; Hayek et al., 2018), one of the structures associated with motor imagery neurofeedback performance in our primary analysis. Although we did not observe any relationship between age and motor imagery neurofeedback performance, we cannot rule out that such a relationship does exist and is mediated by fornix integrity and perhaps general cognitive function. In our sample of older adults, decline of white matter integrity in the fornix may therefore be predictive of motor imagery neurofeedback performance. Our cross-sectional study did not allow us to assess decline of fractional anisotropy in the fornix directly but the observed negative correlation between age and fractional anisotropy in the fornix may provide some support for this hypothesis. In fact, adding age as a predictor for online performance led to the exclusion of fornix fractional anisotropy and the inclusion of fractional anisotropy in the middle cerebellar peduncle, the only region from the set identified by Halder and colleagues that was selected in any of our analyses. However, the observed correlation between fractional anisotropy in the middle cerebellar peduncle and motor imagery neurofeedback performance was negative in our study but positive in the study by Halder and colleagues, so what seems like an overlap between results is, in fact, another difference. Taken together, these results give reason to speculate that differences between younger and older adults in white matter structures might be relevant for motor imagery neurofeedback performance. If this can be confirmed in future studies it would emphasize the necessity of basing prediction models for a particular patient on subjects of similar age.

Finally, another possible explanation for the discrepancy between our results and those from Halder and colleagues is the choice of statistical tests. While the results of both our primary analysis and the classification by Halder and colleagues were highly significant when assessed by binomial test, our permutation analysis suggests that the binomial test overestimates the statistical significance, as has been reported previously (Noirhomme et al., 2014). This suggestion, if true, raises the possibility that the observed associations are coincidental. Although it is generally to be expected that variables selected to predict one outcome measure show somewhat poorer prediction for another outcome measure, the difference in prediction accuracy between online and offline performance in our analyses is also rather large. In this particular case, the difference in prediction accuracy may be further increased by the fact that for individual participants the classifier with the best accuracy may differ between online and offline performance. Nevertheless, the difference is arguably still too large to be explained by these two effects, even in combination. Since there is no reason that offline and online performance should differ in their link to white matter properties, the lack of significant results from our offline analysis supports the interpretation that the observed associations are coincidental.

Given common research practices it is inevitable that a large proportion of studies yield results that cannot be reproduced reliably, even when appropriate methodological and statistical procedures are used (Ioannidis, 2005). This risk may be even larger when machine learning methods are used (Stahl and Pickles, 2018). The cross-validation methods that can be applied in samples of this size are appropriate for model selection, but the nested cross-validation or bootstrapping approaches that are needed to perform model assessment within the same study are not feasible without considerably larger sample sizes (Harrell, 2015; Stahl and Pickles, 2018). To advance the field, independent replication studies are necessary to establish which of the proposed measures generalize to yield significant predictive value in new, unrelated samples. Until this external validation has occurred, any result should be considered tentative. This process requires that properly executed studies using machine learning methods to identify predictors of motor imagery neurofeedback performance are published, regardless of whether they report an initial finding, a non-replication, or a successful replication (Stahl and Pickles, 2018). Once several predictors have been identified which do generalize to new data, these can then be combined to determine which measures independently contribute to prediction.

In summary, we tested whether, as previously reported (Halder et al., 2013), white matter integrity as measured by fractional anisotropy can be used to predict motor imagery neurofeedback performance. While from a conceptual perspective the attempt was successful, the particular areas that contributed to prediction differed markedly from those identified previously. Our results suggest that if predictions are used to determine the potential benefit of motor imagery neurofeedback, it is advisable to base the predictions on data collected using the same paradigm and with subjects whose characteristics match those of the target case as closely as possible. Of course, this conclusion needs to be confirmed in future studies systematically investigating the roles of motor imagery neurofeedback implementation and of age on anatomy-based prediction of motor imagery neurofeedback.

## Data Availability

The datasets generated for this study are available on request to the corresponding author.

## Author Contributions

JM, SD, CZ, and CK contributed to conception and design of the study. JM collected the data. JM, MB, and CK contributed to data processing. JM performed the statistical analysis and wrote the manuscript. All authors contributed to manuscript revision, read, and approved the submitted version.

## Conflict of Interest Statement

The authors declare that the research was conducted in the absence of any commercial or financial relationships that could be construed as a potential conflict of interest.

## Footnotes

1 Halder and colleagues used the term “sensorimotor rhythms brain-computer interface aptitude.”

2 These measures are commonly referred to as classification accuracies, but we refrain from using the term “accuracy” here to avoid confusion with the prediction accuracy discussed later.
